# Lyme Disease in Urban Areas, Chicago

**DOI:** 10.3201/eid1311.070801

**Published:** 2007-11

**Authors:** Dean A. Jobe, Jeffrey A. Nelson, Michael D. Adam, Stephen A. Martin

**Affiliations:** * Gundersen Lutheran Medical Center, La Crosse, Wisconsin, USA; † North Park University, Chicago, Illinois, USA; ‡ Lake County Health Department and Community Health Center, Waukegan, Illinois, USA; § Cook County Department of Public Health, Oak Park, Illinois, USA

**Keywords:** Lyme disease, *Ixodes*, *Borrelia*, Illinois, letter

**To the Editor:** Lyme disease is a multisystem illness caused by infection with the tickborne spirochete *Borrelia burgdorferi*. Most infections in the United States occur in the Northeast and upper Midwest, and the midwestern focus now includes Illinois (*1**,**2*). Previously, the greatest risk of contracting Lyme disease in the Midwest was confined to the northernmost states (Wisconsin and Minnesota) and did not encroach into heavily populated areas around the city of Chicago. However, we showed recently that *B. burgdorferi–*infected *Ixodes scapularis* ticks were recovered from sites in Cook and DuPage counties (*3*), but the percentages of infected ticks were low (<5%). Since that time, however, reports of Lyme disease in Cook County have been reviewed and individual *I. scapularis* tick submissions from Lake County, north of Chicago, have been received. We therefore surveyed new areas north of Chicago (closest was <1 mile from the city limits; farthest was ≈25 miles from the city limits) and examined additional ticks for infection with *B. burgdorferi*.

From December 2006 to May 2007, we collected 172 adult *I. scapularis* ticks from sites to the north and northwest of Chicago (Figure). Adult ticks were collected because nymphal ticks are more difficult to obtain, and the infection rate in adult ticks is similar (*1*). The tick midguts were removed aseptically, inoculated into tubes containing 1 mL of modified Barbour-Stoenner-Kelly medium (*4*), incubated at 35ºC, and examined for spirochetes for up to 3 weeks. Spirochetes were recovered from 21 (32%) of 65 ticks and 40 (37%) of 107 ticks collected from sites in Cook and Lake counties, respectively. In addition, PCR using primers specific for outer surface protein A (*5*) confirmed that the spirochetes were *B. burgdorferi*.

**Figure:**
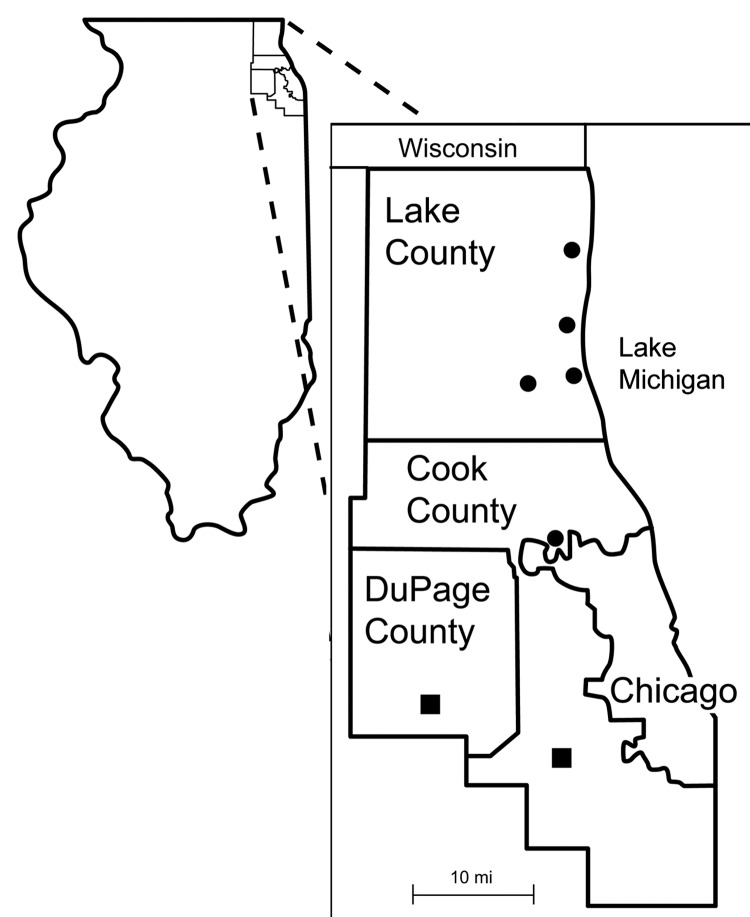
Sites surrounding Chicago from which *Borrelia burgdorferi*–infected *Ixodes scapularis* ticks were recovered in 2005–2006 (■) and 2006–2007 (●).

The findings demonstrate that the midwestern endemic focus of *B. burgdorferi*–infected *I. scapularis* now includes northern Cook and Lake counties. More importantly, the high percentage of *B. burgdorferi*–infected ticks in this region confirms a newly recognized significant risk of Lyme disease in suburban areas adjacent to Chicago (population ≈7 million). Recently, the Infectious Diseases Society of America recommended that clinicians consider prescribing a single prophylactic dose of doxycycline (200 mg) when patients have received tick bites in areas where the percentage of *B. burgdorferi*–infected *I. scapularis* exceeds 20% (*6**,**7*). The high percentage of infected adult ticks identified in this survey highlights the need for physicians in the Chicago area to become familiar with this recommendation, especially considering the high likelihood that nymphal *I. scapularis* ticks are similarly infected (*1*). Moreover, confirmation of the increasing risk of contracting Lyme disease near metropolitan Chicago should provide impetus for more comprehensive studies to completely define the risk of this potentially serious illness.
